# Small mammal species richness and turnover along elevational gradient in Yulong Mountain, Yunnan, Southwest China

**DOI:** 10.1002/ece3.6083

**Published:** 2020-02-23

**Authors:** Zhongzheng Chen, Xueyou Li, Wenyu Song, Quan Li, Kenneth Onditi, Laxman Khanal, Xuelong Jiang

**Affiliations:** ^1^ Anhui Provincial Key Laboratory of the Conservation and Exploitation of Biological Resources College of Life Sciences Anhui Normal University Wuhu China; ^2^ State Key Laboratory of Genetic Resources and Evolution, Kunming Institute of Zoology Chinese Academy of Sciences Kunming China; ^3^ Central Department of Zoology Institute of Science and Technology Tribhuvan University Kathmandu Nepal

**Keywords:** elevational gradients, Hengduan Mountain, small mammals, species turnover, the energy hypothesis, the mid‐domain effect

## Abstract

Understanding the species diversity patterns along elevational gradients is critical for biodiversity conservation in mountainous regions. We examined the elevational patterns of species richness and turnover, and evaluated the effects of spatial and environmental factors on nonvolant small mammals (hereafter “small mammal”) predicted a priori by alternative hypotheses (mid‐domain effect [MDE], species–area relationship [SAR], energy, environmental stability, and habitat complexity]) proposed to explain the variation of diversity. We designed a standardized sampling scheme to trap small mammals at ten elevational bands across the entire elevational gradient on Yulong Mountain, southwest China. A total of 1,808 small mammals representing 23 species were trapped. We observed the hump‐shaped distribution pattern of the overall species richness along elevational gradient. Insectivores, rodents, large‐ranged species, and endemic species richness showed the general hump‐shaped pattern but peaked at different elevations, whereas the small‐ranged species and endemic species favored the decreasing richness pattern. The MDE and the energy hypothesis were supported, whereas little support was found for the SAR, the environmental stability hypothesis, and the habitat complexity. However, the primary driver(s) for richness patterns differed among the partitioning groups, with NDVI (the normalized difference vegetation index) and MDE being the most important variables for the total richness pattern. Species turnover for all small mammal groups increased with elevation, and it supported a decrease in community similarity with elevational distance. Our results emphasized for increased conservation efforts in the higher elevation regions of the Yulong Mountain.

## Graphical abstract

The species richness and turnover pattern for small mammals in Yulong along elevational gradients are demonstrated. The mid‐domain effect (MDE) and the energy hypothesis were supported for most of the small mammal species groups. Species turnover increased with elevation, and it supported a decrease of community similarity with elevational distance.

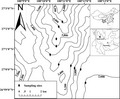

## 1 INTRODUCTION

Mountain systems are key areas of biodiversity which contain a large number of endemic and threatened species and are often recognized as global biodiversity hotspots (Körner & Spehn, 2002; Mittermeier, Turner, Larsen, Brooks, & Gascon, 2011). However, biodiversity in the mountain ecosystems is increasingly under threat because of climate change and human disturbance (Blyth, 2002; Rogora et al., 2018). Understanding the species diversity in mountainous regions is useful for biodiversity conservation and predicting the responses of species to future environmental changes (Fischer, Blaschke, & Bässler, 2011). Elevational gradients can provide equally striking species diversity patterns over short distances, making them very suitable for exploring the mechanisms of spatial variation in diversity (Sanders & Rahbek, 2012).

In the past several decades, studies on the elevational patterns of biodiversity have received increasing attention across continents, and most of them focused on the elevational species richness patterns (McCain & Grytnes, 2010; McCain, King, Szewczyk, & Beck, 2018; Musila et al., 2019). Numerous hypotheses have been proposed to account for species richness patterns along elevational gradients (Lomolino, 2001; Sanders & Rahbek, 2012), but no one currently has unequivocal support. Generally, the mid‐domain effect (MDE; Colwell, Rahbek, & Gotelli, 2004), the species–area relationship (SAR; Rosenzweig, 1995), and the energy hypothesis are the most frequently tested (Colwell et al., 2004; McCain et al., 2018; Sanders & Rahbek, 2012; Wu, Colwell, et al., 2013).

The SAR proposes that larger areas will have more species (Rosenzweig, 1995). The MDE indicates that if species’ ranges are distributed randomly within a bounded domain, more ranges will overlap in the middle of the domain than at the edges which will produce a hump‐shaped pattern of species richness (Colwell & Hurtt, 1994). The energy hypothesis proposes that higher productivity and ambient energy often results in higher species diversity (Hawkins et al., 2003; O'Brien, 2006). The regional energy depends primarily on regional temperature, precipitation, and primary productivity, and a positive energy–richness relationship has been reported by many studies (Evans, Warren, & Gaston, 2005; Luo et al., 2012; McCain, 2007a). In addition, the environmental stability hypothesis, which states that with greater environmental stability, less energy is required for regulatory activities, thereby will increase species richness (Connell & Orias, 1964). Recently, some studies found that the environmental stability hypothesis can explain a substantial proportion of variation in the richness of mammals (Luo et al., 2012) and birds (Gao & Liu, 2018). Plant species richness (PSR) can generate more complex habitats for animals and has also been regarded as a potential driver of small mammal species richness (Wu, Yang, et al., 2013).

Species turnover (i.e., beta diversity) is a measure of the difference in species composition between the sites (Koleff, Gaston, & Lennon, 2003). It is an important component of regional diversity and has consequences for conservation (Socolar, Gilroy, Kunin, & Edwards, 2016). Compared to species richness, the elevational patterns of species turnover have been less studied. Several null models have been used to predict the elevational species turnover patterns. Under the scenario of the mid‐domain model, the lowest value of species turnover will occur in the midelevation area, and a U‐shaped pattern is expected along elevational gradients (Koleff & Gaston, 2001; Mena & Vázquez‐Domínguez, 2005). However, according to Stevens’ rule, the ranges of species might be greater at higher elevations than at lower elevations (Stevens, 1992), so species turnover should decrease at higher altitudes. Currently, only a few empirical studies attempting to explore the elevational species turnover exist, and their results remain controversial (Mena & Vázquez‐Domínguez, 2005).

The Hengduan Mountains (22–32°N, 98–104°E), occurring within an ecotone between the Oriental region and the Palearctic region, are one of the world's biodiversity hotspots (Myers, Mittermeier, Mittermeier, Fonseca, & Kent, 2000). Due to extremely complex topography and variations in habitat, the Hengduan Mountains harbor high species diversity and support a large number of endemic species (Jiang et al., 2015). Despite this, the Hengduan Mountains are considered one of the most threatened mountains because of climate change and human disturbance (Payne, Warrington, & Bennett, 2002). Many elevational studies of species richness and diversity have been conducted in this region, but they show a strong bias toward plants and birds (Feng, Wang, Chengdong, Yang, & Fang, 2006; Luo et al., 2016; Wang, Fang, Sanders, White, & Tang, 2009; Wu, Colwell, et al., 2013). Quantitative studies focusing on elevational patterns of the small mammals in this region are still lacking. In the present study, we conducted an integrative analysis of small mammal species richness and turnover on Yulong Mountain, one of the highest mountains (5,596 m) in the Hengduan Mountains. We trapped small mammals and measured environmental variables at each elevational band. Our aims were to (a) delineate the elevational species richness and turnover patterns of small mammals along Yulong Mountain and (b) to evaluate the relative importance of the five ecological hypotheses described above (MDE, SAR, the energy hypothesis, the environment stability hypothesis, and the habitat complexity hypothesis) in predicting variation of small mammal species diversity along elevational gradient.

## 2 MATERIALS AND METHODS

### 2.1 Ethics approval

The required permissions were obtained from the Lijiang Alpine Botanical Garden, Kunming Institute of Botany. All methods were performed following the guidelines and regulations approved by the internal review board of Kunming Institute of Zoology, Chinese Academy of Sciences (approval ID: SMKX‐2012023).

### 2.2 Study area

**Figure 1:**
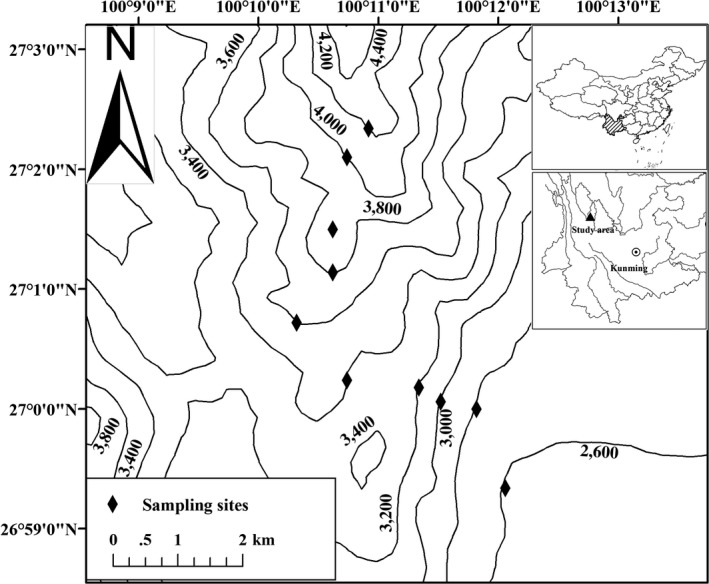
Sketch map of study area in Yulong Mountain, Yunnan, China

The Yulong Mountain (27°10′–27°40′N, 100°09′–100°20′E) is located in the southern part of the Hengduan Mountains, Yunnan Province, southwestern China (Figure 1). It is the southernmost glacierized area in mainland Eurasia which is extremely sensitive to climate change (Pang, Yuanqing, & Zhang, 2007). The mean annual temperature at Yulong Mountain is 12.8°C, and the average annual precipitation is 935 mm (Feng et al., 2006). There is a distinct dry season from November to May and a rainy season from June to October.

The present study was conducted on the southern edge of Yulong Mountain. There is a protected garden of the Lijiang Alpine Botanical Garden of the Kunming Institute of Botany, Chinese Academy of Sciences (CAS), where the forest areas are well protected. In this area, the lowest elevation is about 2,600 m and rocky cliffs dominate the habitats above 4,250 m. The vegetation zonation of this area is apparent along elevational gradients (Luo et al., 2016). There are six major vegetation zones in the area: (a) coniferous forests dominated by *Pinus armandi* (2,600–2,800 m); (b) mixed coniferous/sclerophyllous broad‐leaved forest dominated by *P. armandii* and *P. yunnanensis*, mixed with *Quercus spinosa* (2,800–3,000 m); (c) mixed coniferous and broad‐leaved forest dominated by *Q. guyavifolia* and *P. yunnanensis* (3,000–3,500 m); (d) mixed coniferous and broad‐leaved forest dominated by *Q. aquifolioides*, *Abies georgei*, and *Rhododendron rubiginosum* (3,500–3,900 m); (e) snow mountain meadow habitats dominated by grass and shrub (3,900–4,000 m); and (f) rock–scree belt with sparse grass and shrub (>4,000 m).

### 2.3 Sampling

Small mammals were trapped twice, once during the rainy season (30 August to 28 October 2013) and the next during the dry season (30 March to 29 May 2014). The detail sampling time for each site is shown in Table S1. Because the elevation of the forest edge is about 2,550 m and cliffs dominate the habitats above 4,250 m, we conducted the field surveys from 2,550 to 4,250 m. Normally, sampling sites were established at an elevational interval of 200 m (Figure 1). Because of the unique vegetation (snow mountain meadow), we added an extra sampling site at 3,900 m. Therefore, a total of 10 elevational sampling bands were established: 2,600 m (2,550–2,700 m), 2,800 m (2,700–2,900 m), 3,000 m (2,900–3,100 m), 3,200 m (3,100–3,300 m), 3,400 m (3,300–3,500 m), 3,600 m (3,500–3,700 m), 3,800 m (3,700–3,850 m), 3,900 m (3,850–3,950 m), 4,000 m (3,950–4,100 m), and 4,200 m (4,100–4,250 m) (Figure 1). At each elevational band, six trap lines were established with an interval distance of <50 m between consecutive trap lines to cover the main habitat types (Figure S1). To reduce edge effects, all trap lines were laid out along the middle elevation contour (i.e., 2,600 m and 2,800 m) of each elevation band. Twenty‐five to thirty trap stations were placed every 10 m along each trap line (Figure S1). At each trap station, one Sherman trap and one metallic snap trap were set at 1–2 m apart. In addition, five plastic bucket pitfalls (15 cm in diameter and 20 cm in height) were placed in “likely” spots for shrews along trap lines. The Sherman traps were baited with oat flakes, the snap traps were baited with raw peanuts, whereas no bait was kept in the pitfalls. Traps were checked once in the morning, rebaited, and run for three consecutive nights at each site. In total, around 2,000 trap nights were accumulated at each elevational sampling band. The captured individuals were identified to species, weighed, and measured, and the sex was noted. A limited number of voucher specimens were prepared in stuffed skins with intact skulls. All specimens collected in this study are housed at the Kunming Institute of Zoology, Chinese Academy of Sciences, Yunnan, China.

### 2.4 Ecological variables: MDE, area, climate, productivity, and plant species richness

RangeModel 5 (http://viceroy.eeb.uconn.edu/rangemodel/index.html) was used to calculate the predicted MDE values (Colwell, 2008). As the sampling transects in our study were evenly spaced and discrete, the discrete domain analysis was employed (Colwell, 2008). Because discrete sampling inevitably underestimates the true range size, with some single‐site species had an observed elevational range of zero. To adjust this problem and to avoid the “0 m” range size species being “lost” during the randomization of the midpoints, we added 100 m (half of the distance between two elevational sampling sites) to each end of the recorded range following Brehm, Colwell, and Kluge (2007). For the species with the end of the recorded range at 3,900 m, we added only 50 m to the end of the range. Then, we ran 5,000 randomizations (without replacement) to calculate the mean expected species richness and their 95% confidence intervals for each elevational site (i.e., 2,600 m and 2,800 m).

**Figure 2:**
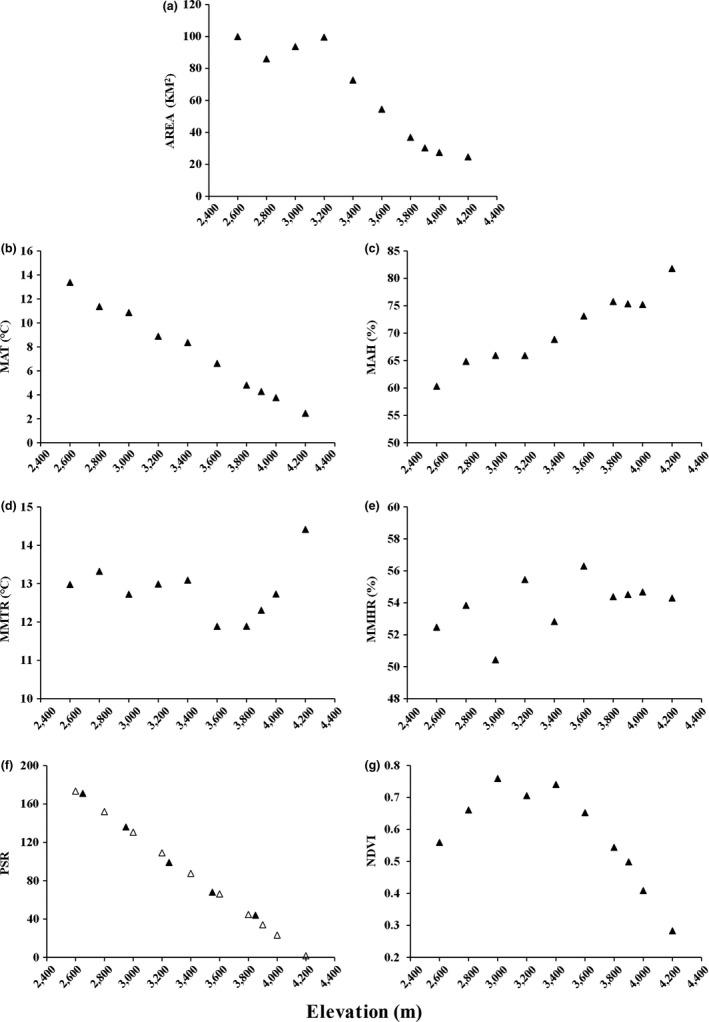
Elevational pattern of Yulong Mountain for (a) area, (b) mean annual temperature (MAT), (c) mean annual humidity, (d) mean monthly temperature range (MMTR), (e) mean monthly humidity range (MMHR), (f) plant species richness (PSR), and (g) productivity (NDVI). Solid triangles in (f) represent observed values, and hollow triangles are predicted values estimated using a linear model

We divided the range of Yulong Mountain into 200‐m elevational bands from 2,500 m to 4,300 m (e.g., 2,500–2,699 m and 2,700–2,899 m). The planimetric area of each elevational band was calculated for each sampling site (e.g., 2,500–2,699 m for the sampling site 2,600 m, Figure S1) using a 30‐m resolution digital elevation in ArcGIS 10.3 and ENVI 5.2. The data were downloaded from the United States Geological Survey (USGS). The elevational trends of the area are shown in Figure 2.

To obtain accurate climatic data, we installed two temperature and humidity data loggers (DS1923, Thermochron^®^ iButton^®^; San Jose Semiconductor/Maxim) at each elevational band. Data loggers were installed near tree trunks in order to avoid direct sunlight and set to record temperature and humidity at intervals of 2 hr for a whole year (from 1 November 2013 to 31 October 2014). Four climatic factors that may contribute to the species richness patterns of small mammals were investigated in our analysis: mean annual temperature (MAT), mean annual humidity (MAH), mean monthly temperature range (MMTR), and mean monthly humidity range (MMHR).

We used the normalized difference vegetation index (NDVI) as a surrogate for primary productivity. NDVI was calculated at each elevational gradient using Landsat 8 OLI digital image downloaded from the United States Geological Survey (USGS). A 5‐km rectangular plot of each transect was extracted using the software ENVI 5.2. The NDVI was calculated for each elevational band based on these plots using the formula [NDVI = (ρ_nir_ − ρ_red_)/(ρ_nir_ + ρ_red_)], where ρ_nir_ = near‐infrared band and ρ_red_ = visible red band.

The plant species richness (PSR), including woody, shrub, and herbaceous species, was measured by Luo et al. (2016) at five elevations (2,650 m, 2,950 m, 3,250 m, 3,550 m, and 3,850 m) along the same slope from May to August 2013. According to their data, plant species richness decreases with increasing altitude at a rate of 10.73 species/100 m (Figure 2f; *R* ^2^ = 0.99, *p* < .001). We estimated PSR for our sampling sites by using a linear model based on the records in the five elevations.

### 2.5 Data analysis

Previous research showed that species richness patterns and their causes vary among different taxonomic groups (Chen, He, Cheng, Khanal, & Jiang, 2017), range size (Dunn, McCain, & Sanders, 2007), and endemism (Colwell et al., 2004; Fu et al., 2006). To assess these predictions, we divided the total small mammal species into insectivores and rodents, large‐ranged and small‐ranged species, and endemic and nonendemic species. Large‐ranged species were defined as the half of the species with elevational distribution ranges larger than the median size; the others were considered to be small‐ranged species. Endemic species refer to those that only occur in the Hengduan Mountains and the eastern Himalaya region. We repeated all the downstream analyses separately for total species and the six subsets of small mammal species.

We considered each elevational band as one sampling unit for data analysis. On each elevational band, we pooled the occurrence data of the six trap lines of both seasons and treated it as a “community.” Species richness was obtained by counting the number of all species recorded by the six trap lines. The potential for pseudoreplication in our study was limited by pooling all trap lines for a given elevation band. Polynomial regressions were used to estimate the relationship between species richness, turnover, and elevation for each species group, guided by the corrected Akaike information criterion (AICc) value.

An information‐theoretic approach (Anderson, Burnham, & Thompson, 2000; Stephens, Buskirk, Hayward, & Martinez Del Rio, 2005) was used to evaluate the relative importance of the mid‐domain effect, the species–area relationship, the energy hypothesis, the environment stability hypothesis, and the habitat complexity hypothesis to small mammals. First, we log‐transformed the eight potential drivers (MDE, area, MAT, MAH, MMTR, MMHR, NDVI, and PSR) to obtain the normality and homoscedasticity of the data. Then, we used the generalized linear model to develop a set of candidate models based on a priori hypotheses. Owing to the complication of energy theory, we establish the models that included all the possible combinations of three energy‐related factors (MAT, MAH, and NDVI). For comparison, a null model (species ~ 1) was added in our analysis. We calculated the variance inflation factor (VIF) of each variable in these models to account for collinearity. To minimize the multicollinearity, only the models with VIFs < 10 were considered (Dormann et al., 2013). Finally, a set of 10 candidate models were retained for further analysis (Table S2). Then, we selected the best model based on the lowest AICc value using the R package “MuMIn” (Bartoń, 2015). To prevent other interesting models from being ignored, all ten models for each species group with their adjust *R* ^2^, AICc, ΔAICc, and AICc weights are listed in Tables S1. Two or more nearly equivalent supported models (i.e., ΔAICc ≤ 2) were observed for the total species, large‐ranged species, and small‐ranged species groups (Table 2). We performed model averaging to evaluate the relative importance of each variable in shaping the elevational richness patterns of these groups (Claeskens & Hjort, 2008; Galipaud, Gillingham, & Dechaume‐Moncharmont, 2017). We inferred strong support for an effect whenever 95% confidence intervals (CI) for model‐averaged effects excluded zero (Grueber, Nakagawa, Laws, & Jamieson, 2011).

We calculated the Sørensen similarity index based on the abundance data of each species to measure species turnover between pairwise elevational transects in EstimateS 9.1.0 (Colwell, 2013). We first used small mammal communities between the neighboring transects (i.e., 2,600 m vs. 2,800 m; and 2,800 m vs. 3,000 m) to analyze species turnover along elevational gradients. Again, polynomial regressions were used to estimate the elevational species turnover patterns of each small mammal group. Then, we plotted the similarity index of all paired elevations against changes in elevational distance. The relationship between Sørensen similarity and elevational distance was determined by fitting a linear regression. We reported the slope with 95% confidence intervals of each linear regression to reflect the beta diversity of each small mammal group (Harte, Kinzig, & Green, 1999).

## 3 RESULTS

### 3.1 Species richness pattern

**Figure 3:**
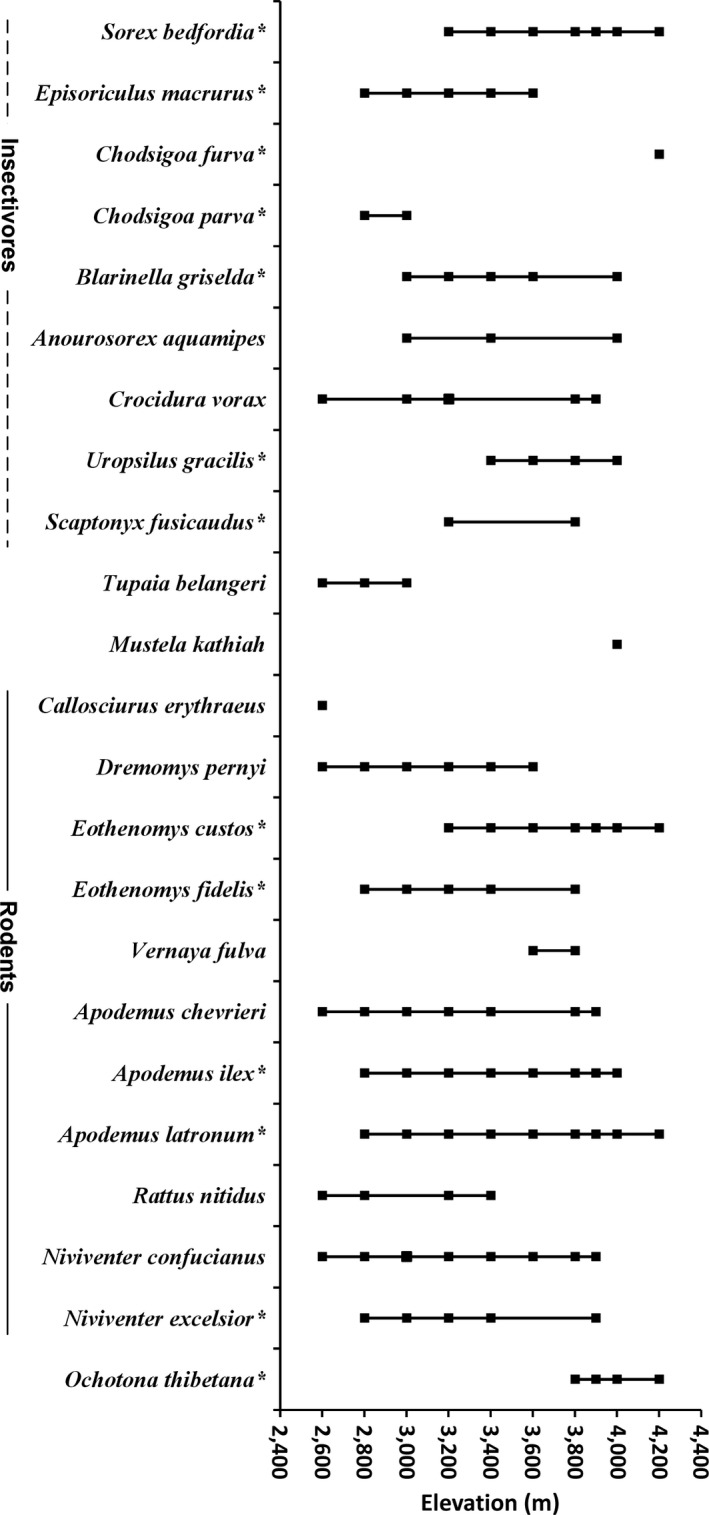
Elevational distribution range, taxonomy, and endemism of each small mammal species in the Yulong Mountain. Solid squares indicate the elevation at which individuals were trapped. *Endemic species

A total of 1808 individuals representing 23 species under 18 genera of small mammals were trapped over 20,420 trap nights, with a total trap success rate of 8.85%. The number of individuals captured per elevational transect varied from 65 to 318, and the species varied from 5 to 14. Among the 23 species, 9 were insectivores and 11 were rodents; 13 were endemic and 10 were nonendemic species (Figure 3).

**Figure 4:**
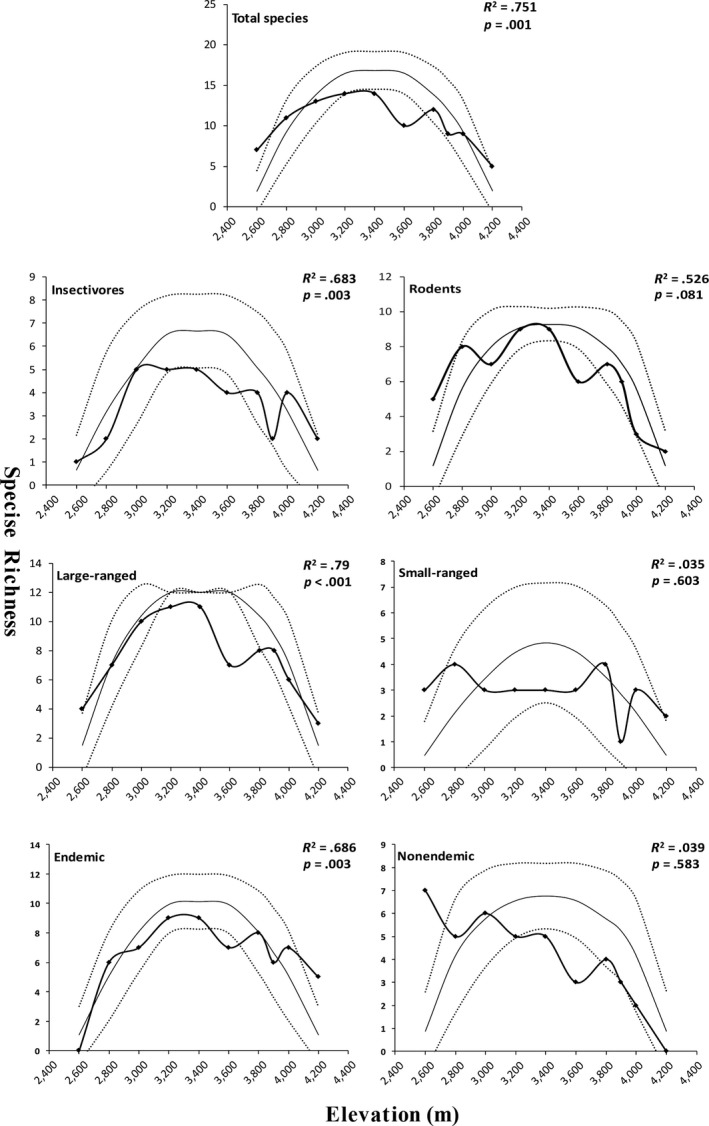
Species richness curves of small mammals (line with squares) along the elevation gradient of the Yulong Mountain. The MDE‐null predicted species richness (lines only) with 95% confidence intervals (dotted lines) is based on 5,000 simulations samples in RangeModel 5. The *R* ^2^ and *p*‐values were obtained by doing a linear regression of the observed richness on the predicted values to estimate the impact of the null model

Most small mammal groups in the Yulong Mountain showed a hump‐shaped richness pattern along the elevational gradient except small‐ranged species, and nonendemic species decreased as the elevation increased (Figure 4). This result was confirmed by the polynomial regression analysis (Table 1).

**Table 1:** Polynomial regressions of species richness and Sørensen similarity index of different small mammal groups along elevational gradients in the Yulong Mountain

	*K* a	Total species	Insectivores	Rodents	Small‐ranged species	Small‐ranged species	Endemic species	Nonendemic species
Species richness								
Linear	3	58.12	45.42	51.03	56.88	**32.31**	55.06	**34.00**
Quadratic	4	**45.33**	**39.61**	**42.33**	**44.76**	37.82	**45.73**	36.61
Cubic	5	52.62	45.87	51.18	51.77	46.81	46.87	42.00
Sørensen similarity								
Linear	3	**−1.38**	**11.34**	**2.76**	**1.06**	**9.37**	**−2.62**	**7.74**
Quadratic	4	1.41	9.77	10.93	5.04	16.11	11.27	16.54
Cubic	5	12.84	26.47	21.06	3.97	32.17	53.05	54.68

Note

Bold letters indicate the best model based on the lowest corrected Akaike information criterion (AICc) values.

a Number of parameters.

### 3.2 Relationship between species richness and explanatory factors

The information‐theoretic statistics for the ten candidate models showed that the best‐fit model varied among different species groups (Table 2). Nevertheless, in most data sets, MDE and/or the energy‐related models were suggested as the best model. MDE is the best model for total species richness, which had an Akaike weight (*W*_i_) of 0.37. Two alternative energy‐related models with only slightly higher AICc (ΔAICc ≤ 2), one including only NDVI (*W*_i_ = 0.25), and the other including NDVI and MAT (*W*_i_ = 0.27), were available. All the three models explained a substantial proportion of variation for total species richness pattern (*R* ^2^ > 0.70, *p* < .01). The MDE model was also suggested as the best model for the richness patterns of insectivores (*W*_i_ = 0.81), large‐ranged species (*W*_i_ = 0.57), an alternative model including NDVI and MAT (ΔAICc = 1.35; *W*_i_ = 0.29), and endemic species (*W*_i_ = 0.79). The model included only NDVI as the best model for rodents (*W*_i_ = 0.82). The model included only MAT was supported as the best model for the richness patterns of nonendemic species (*W*_i_ = 0.60). The null model (richness ~ 1) had the lowest AICc for small‐ranged species (*W*_i_ = 0.33). However, the ΔAICc of the top seven ranked models was only 2.53, suggesting the uncertainty of the best model (Table S2). It should be noted that despite the extensive sampling effort across the entire elevational gradient, the sample size for multiple regressions is small (*n* = 10); the results in Table 2 should, therefore, be considered exploratory rather than definitive.

**Table 2:** Results of model selection (best model) for the richness of different small mammal species groups along elevational gradients in Yulong Mountain

Model	Hypothesis	*K* a	Adjust *R* ^2^	AICc	ΔAICc	AICc *W*_i_
Total species						
MDE	Mid‐domain effect	3	0.72**	46.52	0.00	0.37
NDVI	Energy hypothesis	3	0.70**	47.33	0.82	0.25
NDVI + MAT	Energy hypothesis	4	0.82**	47.17	0.65	0.27
Insectivores						
MDE	Mid‐domain effect	3	0.61**	36.13	0.00	0.81
Rodents						
NDVI	Energy hypothesis	3	0.80***	38.41	0.00	0.82
Large‐ranged species						
MDE	Mid‐domain effect	3	0.74**	44.01	0.00	0.57
NDVI + MAT	Energy hypothesis	4	0.81**	45.36	1.35	0.29
Small‐ranged species						
1	Null model	2	n.a.	30.38	0.00	0.33
AREA	Species–area relationship	3	0.21	32.35	1.97	0.12
PSR	Habitat complexity hypothesis	3	0.23	32.04	1.66	0.14
MAT	Energy hypothesis	3	0.23	32.10	1.72	0.14
Endemic species						
MDE	Mid‐domain effect	3	0.71**	43.99	0.00	0.79
Nonendemic species						
MAT	Energy hypothesis	3	0.90***	29.24	0.00	0.60

Abbreviations: MAT, mean annual temperature; MDE, the mid‐domain effect; MMHR, mean monthly temperature range; MMTR, mean monthly temperature range; NDVI, normalized difference vegetation index; PSR, plant species richness.

a Number of parameters.

** *p* < .01, ***p < .001.

The model averaging results showed that only MDE and NDVI with a 95% confidence interval did not overlap zero for both the total species and large‐ranged species (Table 3), suggesting a substantial effect. NDVI was supported as the most important factor for the two species groups, as revealed by the higher beta coefficients. All confidence intervals of the parameter estimate for small‐ranged species overlapped zero, suggesting a weak effect for all of the predictor variables.

**Table 3:** Estimate, unconditional standard errors (*SE*), and 95% confidence interval (CI) of each variable determined by model averaging in shaping the elevational richness pattern for the total species, large‐ranged species, and small‐ranged species in Yulong Mountain

	Total species			Large‐ranged species			Small‐ranged species		
	Estimate	*SE*	95% CI	Estimate	*SE*	95% CI	Estimate	*SE*	95% CI
(Intercept)	0.00	0.00	0.00–0.00	0.00	0.00	0.00–0.00	0.00	0.00	0.00–0.00
MDE	**0.85**	**0.22**	**0.42**–**1.28**	**0.86**	**0.21**	**0.44**–**1.28**	0.48	0.36	−0.23–1.20
NDVI	**1.14**	**0.39**	**0.37**–**1.90**	**1.34**	**0.43**	**0.49**–**2.18**	0.47	0.39	−0.30–1.24
MAT	−0.67	0.36	−1.37–0.03	−0.87	0.35	−1.56–0.18	0.45	0.37	−0.27–1.18
MAH	0.44	0.31	−0.16–1.04	0.54	0.34	−0.13–1.20	0.41	0.42	−0.42–1.24
PSR	0.63	0.32	0.00–1.26	0.54	0.35	−0.14–1.23	−0.39	0.40	−1.17–0.39
AREA	0.54	0.35	−0.15–1.23	0.45	0.37	−0.28–1.18	0.21	0.41	−0.58–1.01
MMTR	−0.43	0.42	−1.25–0.39	−0.41	0.42	−1.24–0.42	−0.19	0.46	−1.09–0.70
MMHR	−0.25	0.42	−1.07–0.57	−0.24	0.42	−1.07–0.59	−0.14	0.46	−1.03–0.76

Note

Estimates with confidence intervals that do not overlap zero are shown in bold.

Abbreviations: MAT, mean annual temperature; MDE, the mid‐domain effect; MMHR, mean monthly temperature range; MMTR, mean monthly temperature range; NDVI, normalized difference vegetation index; PSR, plant species richness.

### 3.3 Species turnover along elevational gradients

**Figure 5:**
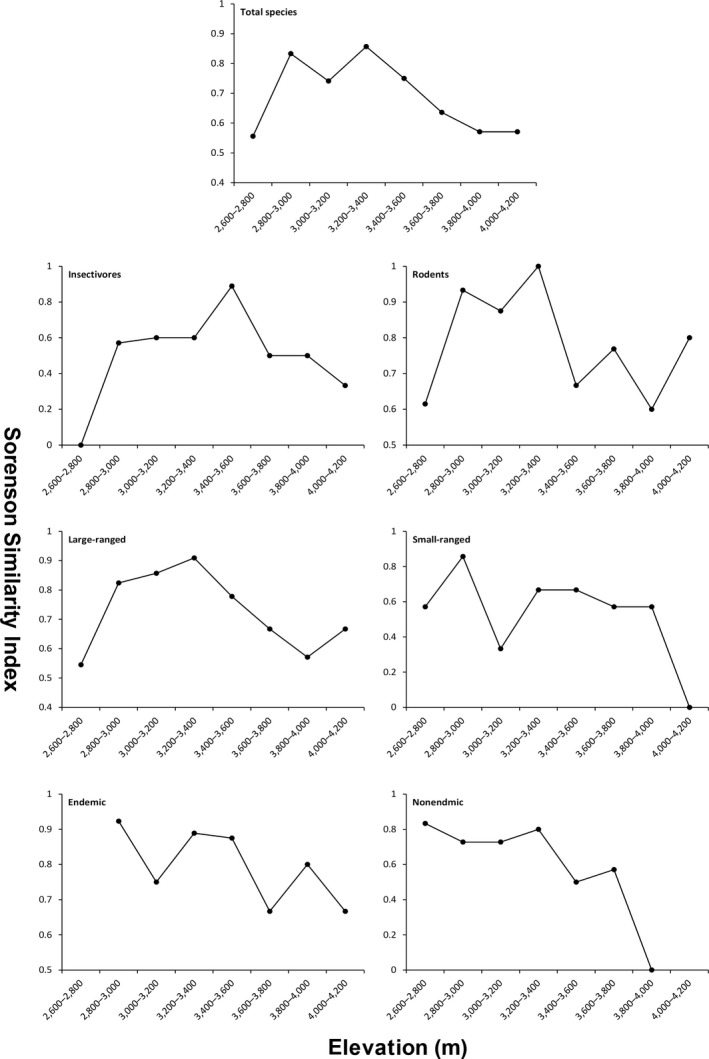
The Sørensen similarity index for small mammals between adjacent elevational bands in Yulong Mountain

**Figure 6:**
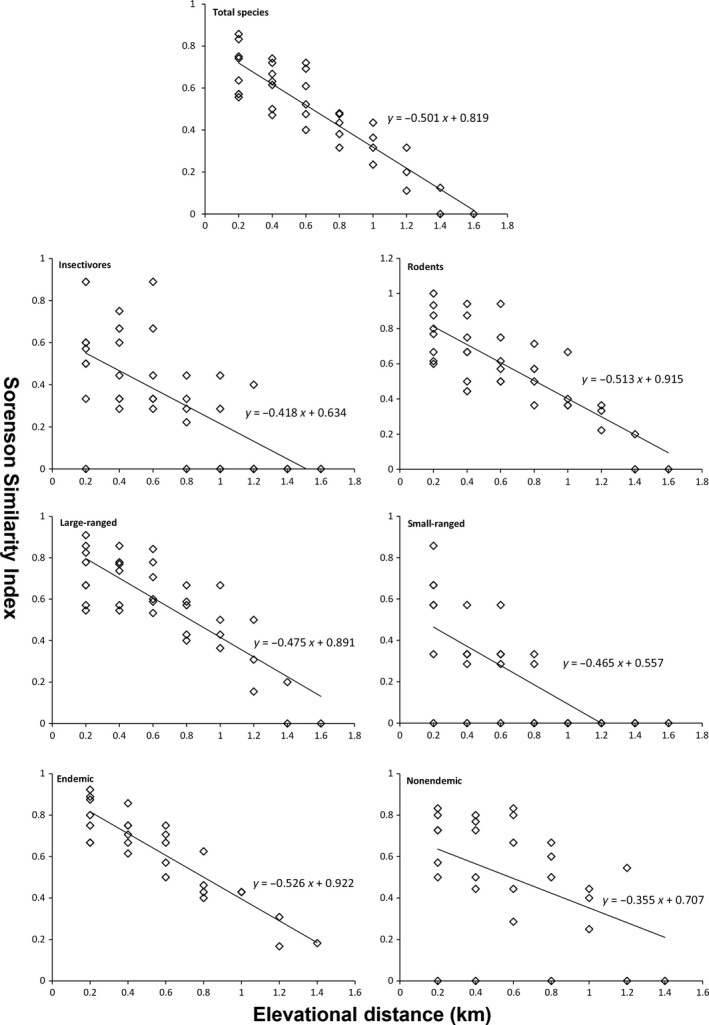
Variation in Sørensen similarity index with different elevational distance for small mammals in Yulong Mountain. Lines represent the relation trend. Rhombus indicate the data points of Sorensen similarity index vs. elevational distance

A high level of species turnover at the base of the Mountain was observed for the total species, insectivores, rodents, and large‐ranged species (Figure 5). The polynomial regression of Sørensen similarity along elevational gradients showed that all species groups are better fit by a linear regression than a quadratic or cubic function of elevation (Table 1). For all species groups, the Sørensen similarity index at higher elevations (>3,600 m) is low indicating a high beta diversity. There is a consistent decreasing pattern of Sørensen similarity with elevational distance for all species groups (Figure 6; indicating a distance–decay of similarity in communities). The rate of decline in similarity was not significantly different among different species groups (Table S3).

## 4 DISCUSSION

By using detailed field data and integrative analyses, our results are the first to rigorously document small mammal species richness patterns along elevational gradients in Yulong Mountain. The overall species richness of small mammal exhibited a hump‐shaped pattern along the elevational gradients, with a distinct peak at 3,200 m and 3,400 m (Table 1; Figure 4). Such a pattern is frequently reported in the Hengduan Mountains for frogs (Fu et al., 2006), birds (Wu, Colwell, et al., 2013), as well as small mammals (Chen et al., 2017). These results suggest the hump‐shaped pattern might be the general richness pattern in the Hengduan Mountains.

Our analysis showed that MDE and the energy hypothesis concur to the elevational species richness of small mammals. Model selection among alternative models for the overall species is nearly equivalent in support of the two hypotheses (ΔAICc < 2; Table 2). MDE and energy‐related factor NDVI were further confirmed as the only two important factors by model averaging (Table 3). When the total species are divided into different subsets, the MDE and the energy‐related models were also supported as the best model(s) for three and four of the six subsets of small mammal groups, respectively (Table 2). It is not surprising that the energy hypothesis is supported in our data, as a positive relationship between diversity and productivity of small mammals was frequently reported by previous studies (Chen et al., 2017; McCain et al., 2018). MDE has generated considerable controversy because of the high variable explanatory power (McCain, 2007b), even in studies on small mammals (Hu et al., 2017; Rowe, 2009; Wu, Yang, et al., 2013). A possible reason for the high fit of MDE in our study is the vast extent of our study area (2600–4200 m), which makes a high degree of overlap in the central area. Determining the circumstances under which MDE works is an essential issue for biogeography (Dunn et al., 2007). In contrast, the support for SAR, the environmental stability hypothesis, and the habitat complexity hypothesis by our data were low when compared with the alternative ones (Table S2).

We found that the primary driver(s) of species richness varied among different subsets of species (Tables 2 and 3), suggesting a multiple causal framework for the elevational richness patterns. Our results supported the hypothesis that large‐ranged species are better fitted to MDE than small‐ranged species (Dunn et al., 2007). However, we found that the impact of NDVI on large‐ranged species was even more significant than MDE (Table 3) that might indicate more complicated underlying mechanisms for the pattern of large‐ranged species richness. Endemic species peaked at midelevations and was best explained by MDE, whereas the nonendemic species richness peaked at low elevations explained mostly by MAT (Table 2). These results are consistent with the inference that the distributions of endemic species are more affected by geometric constraints than more widespread nonendemic species (Colwell et al., 2004; Dunn et al., 2007), whereas the decreasing pattern of nonendemic mammal species richness may be due to the filtering of the harsh climate (e.g., lower temperatures) on the high elevations (Nogué, Rull, & Vegas‐Vilarrúbia, 2013). Recent studies showed that NDVI is the strongest driver for the richness of insectivores, with area and MDE as most important factors for rodents (Chen et al., 2017). However, in our study, the insectivore species richness was mostly affected by MDE while NDVI most influenced that of rodents. The differing results indicate that the primary drivers of insectivores and rodents might depend on where we study them. Different influence factor(s) in each subgroup may result in complex underlying drivers for the overall species. Broader comparative studies within or between species groups, as well as improved knowledge of the life histories of small mammals, should be highlighted to enhance our understanding of the elevational richness patterns and their underlying mechanisms (Graham et al., 2014; Lomolino, 2001; Rahbek, 2005).

There was an evident species turnover of small mammals with elevation in Yulong Mountain (Figure 5). According to the assumption of Stevens' rule, species turnover will decrease at high elevations because species range size increases at higher elevations (Stevens, 1992). In our study, we found little evidence to support Stevens' rule for small mammals in Yulong Mountain. On the contrary, we found a high species turnover in the higher elevations, and the mean range size of high‐elevation species is even smaller than low‐elevation species (755 m for species with range midpoints >3,400 m, while 983 m for species with range midpoints ≤3,400 m). We observed higher values of species turnover at the base of Yulong Mountain for total species, which may relate to human disturbance. Around 2,600 m, human activity may have created a suitable habitat for species associated with humans (e.g., *Apodemus chevrieri*). Despite the polynomial regression analyses suggested a linear relationship, the highest Sørensen similarity of total species as well as insectivores, rodents, and large‐ranged species with elevation occurs at intermediate elevations (Figure 5), somewhat supports the predictions of the mid‐domain model (Koleff & Gaston, 2001). A consistent pattern of distance–decay relationship of community similarity was found across all species groups (Figure 6). Such results suggest a significant influence of dispersal limitation in shaping small mammal distribution patterns in Yulong Mountain (Soininen, McDonald, & Hillebrand, 2007).

The Yulong Mountain has a very high small mammal species diversity and supports a high percentage of endemic species (Figure 3; Jiang et al., 2015). However, as the southernmost snow mountain in the northern hemisphere, it is susceptible to climate change (Pang et al., 2007). The heavily developed tourism industry of Yulong Mountain makes it one of the most threatened mountains in the world (Payne et al., 2002). In our study, area remaining only as a protected garden of CAS which covers only the lower‐middle elevations (<3,600 m) is insufficient for conservation, especially in the higher elevations. First, our results showed high species turnover in the higher elevations (Figure 5). However, these areas face many conservation challenges from human activities including grazing and logging. What is more, studies have demonstrated that climate warming can cause small mammals to expand their range upward in montane regions, making species at high altitudes more vulnerable (Moritz et al., 2008). In the Yulong Mountain, the endemic small mammals tend to be distributed at high elevations (Figures 2 and 4); if global warming continues, the endemic species will be much threatened. Thus, our results strongly support the idea that the protected area should cover the higher elevations, and long‐term monitoring of the small mammals in this area would be valuable to identify how small mammal diversity respond to climate change.

## CONFLICT OF INTEREST

The authors declare there are no competing interests.

## AUTHOR CONTRIBUTION

Z.C. and X.J. designed and performed the study. X.L., Q.L., W.S., K.O., and L.K. contributed to the fieldwork. Z.C. performed analyses and led the writing. X.J., X.L., Q.L., W.S., K.O., and L.K. reviewed drafts of the paper. All authors read and approved the final version of the manuscript.

## Data Availability

Sampling locations, species richness and abundance, and ecological variables data: Dryad https://doi.org/10.5061/dryad.0gb5mkkxm
