# Supplementary material for: Small mammal species richness and turnover along elevational gradient in Yulong Mountain, Yunnan, Southwest China
Source: Ecol Evol. 2020 Feb 23;10(5):2545–58. doi: 10.1002/ece3.6083 (PMC7069287; doi:10.1002/ece3.6083)
Supplement: Supplementary file 1 [file ECE3-10-2545-s001.docx]

**Small mammal species richness and turnover along elevational gradient in Yulong Mountain, Yunnan, southwest** **China**

**Supplementary Files**


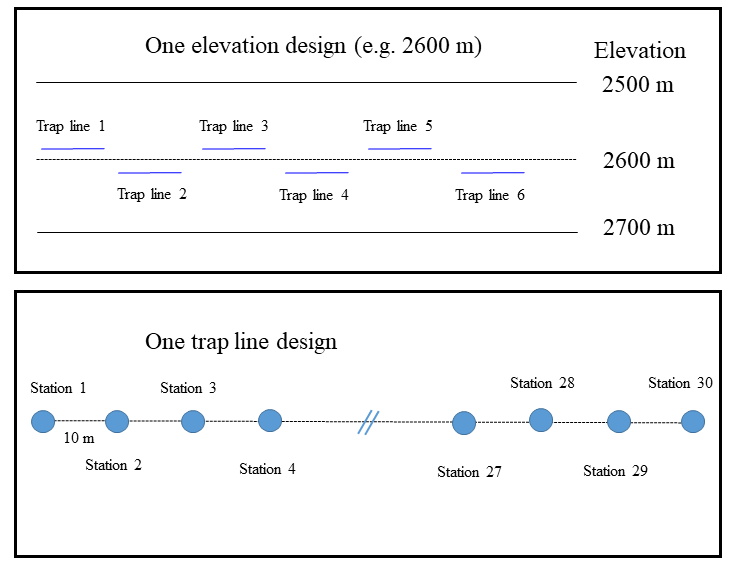


**Figure S1.** Sketch map of the sampling design for an elevational band.

Table S1. Sampling time at each elevation site

| Sampling Date | Days | Seasons | Elevations(m) |
| --- | --- | --- | --- |
| 2013.8.30-9.1 | 3 | rainy season | 3600 |
| 2013.9.2-9.7 | 6 | rainy season | 3800 |
| 2013.9.8-9.10 | 3 | rainy season | 3900 |
| 2013.9.11-9.16 | 6 | rainy season | 4000 |
| 2013.9.17-9.22 | 6 | rainy season | 4200 |
| 2013.9.23-9.25 | 3 | rainy season | 3900 |
| 2013.9.26-9.28 | 3 | rainy season | 3600 |
| 2013.9.29-10.4 | 6 | rainy season | 3400 |
| 2013.10.5-10.10 | 6 | rainy season | 3200 |
| 2013.10.11-10.16 | 6 | rainy season | 2600 |
| 2013.10.17-10.22 | 6 | rainy season | 2800 |
| 2013.10.23-10.28 | 6 | rainy season | 3000 |
| 2014.3.30-4.1 | 3 | dry season | 3900 |
| 2014.4.3-4.8 | 6 | dry season | 3600 |
| 2014.4.9-4.14 | 6 | dry season | 4200 |
| 2014.4.15-4.20 | 6 | dry season | 4000 |
| 2014.4.21-4.23 | 3 | dry season | 3900 |
| 2014.4.24-4.29 | 6 | dry season | 3800 |
| 2014.4.30-5.5 | 6 | dry season | 3400 |
| 2014.5.6-5.11 | 6 | dry season | 3200 |
| 2014.5.12-5.17 | 6 | dry season | 3000 |
| 2014.5.18-5.23 | 6 | dry season | 2800 |
| 2014.5.24-5.29 | 6 | dry season | 2600 |

Table S2 Candidate models explaining variation for richness patterns of small mammals in Yulong Mountain.

|  |  | Total species | | | | Insectivores | | | | Rodents | | | |
| --- | --- | --- | --- | --- | --- | --- | --- | --- | --- | --- | --- | --- | --- |
| Model | K^a^ | Adjust R^2^ | AICc | ΔAICc | AICc wi | Adjust R^2^ | AICc | ΔAICc | AICc wi | Adjust R^2^ | AICc | ΔAICc | AICc wi |
| Richness ~1 | 2 | n.a. | 54.94 | 8.42 | 0.01 | n.a. | 41.22 | 5.10 | 0.06 | n.a. | 50.11 | 11.69 | 0.00 |
| **Mid-domain effect** | | |  |  |  |  |  |  |  |  |  |  |  |
| MDE | 3 | **0.72**** | **46.52** | **0.00** | **0.37** | **0.61**** | **36.13** | **0.00** | **0.81** | 0.53* | 48.06 | 9.65 | 0.01 |
| **Species-area relationship** | |  |  |  |  |  |  |  |  |  |  |  |  |
| AREA | 3 | 0.29 | 55.77 | 9.25 | 0.00 | 0.04 | 45.13 | 9.00 | 0.01 | 0.48* | 47.80 | 9.39 | 0.01 |
| **Habitat complexity hypothesis** | | |  |  |  |  |  |  |  |  |  |  |  |
| PSR | 3 | 0.40* | 54.11 | 7.59 | 0.01 | 0.05 | 45.02 | 8.90 | 0.01 | 0.56* | 46.22 | 7.81 | 0.02 |
| **Environmental stability hypothesis** | | |  |  |  |  |  |  |  |  |  |  |  |
| MMTR+MMHR | 4 | 0.10 | 62.98 | 16.46 | 0.00 | 0.00 | 50.34 | 14.22 | 0.00 | 0.02 | 59.01 | 20.60 | 0.00 |
| **Energy hypothesis** | | |  |  |  |  |  |  |  |  |  |  |  |
| MAT | 3 | 0.24 | 56.44 | 9.92 | 0.00 | 0.01 | 45.43 | 9.30 | 0.01 | 0.44* | 48.53 | 10.12 | 0.01 |
| MAH | 3 | 0.13 | 57.82 | 11.30 | 0.00 | 0.00 | 45.50 | 9.37 | 0.01 | 0.23 | 50.93 | 12.52 | 0.00 |
| NDVI | 3 | **0.70**** | **47.33** | **0.82** | **0.25** | 0.25 | 42.66 | 6.54 | 0.03 | **0.80***** | **38.41** | **0.00** | **0.82** |
| NDVI+MAT | 4 | **0.82**** | **47.17** | **0.65** | **0.27** | 0.56* | 42.11 | 5.98 | 0.04 | 0.80** | 43.25 | 4.84 | 0.07 |
| NDVI+MAH | 4 | 0.77** | 49.25 | 2.73 | 0.09 | 0.48 | 43.72 | 7.59 | 0.02 | 0.79** | 43.60 | 5.19 | 0.06 |

a: Number of parameters. *P < 0.05; **P < 0.01; ***P < 0.001. Bold letters indicate the models with ΔAICc ≤ 2. MDE, the mid-domain effect; MAT, mean annual temperature; NDVI, normalized difference vegetation index; PSR, plant species richness; MMTR, mean monthly temperature range; MMHR, mean monthly temperature range.

|  |  | Large-ranged species | | | | Small-ranged species | | | | Endemic species | | | | Non-endemic species | | | |
| --- | --- | --- | --- | --- | --- | --- | --- | --- | --- | --- | --- | --- | --- | --- | --- | --- | --- |
| Model | K^a^ | Adjust R^2^ | AICc | ΔAICc | AICc wi | Adjust R^2^ | AICc | ΔAICc | AICc wi | Adjust R^2^ | AICc | ΔAICc | AICc wi | Adjust R^2^ | AICc | ΔAICc | AICc wi |
| Richness ~1 | 2 | n.a. | 53.04 | 9.03 | 0.01 | n.a. | **30.38** | **0.00** | **0.33** | n.a. | 52.08 | 8.09 | 0.01 | n.a. | 47.44 | 18.21 | 0.00 |
| **Mid-domain effect** | | |  |  |  |  |  |  |  |  |  |  |  |  |  |  |  |
| MDE | 3 | **0.74**** | **44.01** | **0.00** | **0.57** | 0.05 | 34.20 | 3.82 | 0.05 | **0.71**** | **43.99** | **0.00** | **0.79** | 0.03 | 51.44 | 22.21 | 0.00 |
| **Species-area relationship** | | |  |  |  |  |  |  |  |  |  |  |  |  |  |  |  |
| AREA | 3 | 0.20 | 55.09 | 11.07 | 0.00 | **0.21** | **32.35** | **1.97** | **0.12** | 0.01 | 56.29 | 12.30 | 0.00 | 0.80*** | 35.88 | 6.64 | 0.02 |
| **Habitat complexity hypothesis** | | |  |  |  |  |  |  |  |  |  |  |  |  |  |  |  |
| PSR | 3 | 0.29 | 53.86 | 9.85 | 0.00 | **0.23** | **32.04** | **1.66** | **0.14** | 0.00 | 56.36 | 12.37 | 0.00 | 0.85*** | 33.01 | 3.78 | 0.09 |
| **Environmental stability hypothesis** | | | |  |  |  |  |  |  |  |  |  |  |  |  |  |  |
| MMTR+MMHR | 4 | 0.08 | 61.33 | 17.32 | 0.00 | -0.08 | 40.22 | 9.83 | 0.00 | -0.01 | 61.26 | 17.27 | 0.00 | 0.37 | 51.89 | 22.66 | 0.00 |
| **Energy hypothesis** | | |  |  |  |  |  |  |  |  |  |  |  |  |  |  |  |
| MAT | 3 | 0.15 | 55.68 | 11.67 | 0.00 | **0.23** | **32.10** | **1.72** | **0.14** | 0.03 | 56.03 | 12.04 | 0.00 | **0.90***** | **29.24** | **0.00** | **0.60** |
| MAH | 3 | 0.07 | 56.58 | 12.56 | 0.00 | 0.16 | 32.91 | 2.53 | 0.09 | 0.10 | 55.28 | 11.29 | 0.00 | 0.87*** | 31.63 | 2.39 | 0.18 |
| NDVI | 3 | 0.61** | 48.04 | 4.02 | 0.08 | 0.19 | 32.60 | 2.22 | 0.11 | 0.10 | 55.28 | 11.29 | 0.00 | 0.65** | 41.13 | 11.89 | 0.00 |
| NDVI+MAT | 4 | **0.81**** | **45.36** | **1.35** | **0.29** | 0.13 | 38.05 | 7.67 | 0.01 | 0.75** | 47.28 | 3.29 | 0.15 | 0.88*** | 35.15 | 5.91 | 0.03 |
| NDVI+MAH | 4 | 0.73** | 49.00 | 4.99 | 0.05 | 0.11 | 38.37 | 7.99 | 0.01 | 0.66* | 50.35 | 6.36 | 0.03 | 0.90** | 33.42 | 4.19 | 0.07 |

Table S2. Continued

a: Number of parameters. *P < 0.05; **P < 0.01; ***P < 0.001. Bold letters indicate the models with ΔAICc ≤ 2. MDE, the mid-domain effect; MAT, mean annual temperature; NDVI, normalized difference vegetation index; PSR, plant species richness; MMTR, mean monthly temperature range; MMHR, mean monthly temperature range.

Table S3 Results of the linear regression for the distance decay relationships of community similarity along elevational gradients of small mammals in Yulong Mountain.

| Species groups | Estimate | SE | 2.5% CI | 97.5% CI | P value |
| --- | --- | --- | --- | --- | --- |
| Total species | -0.501 | 0.033 | -0.588 | -0.414 | <0.001 |
| Insectivores | -0.418 | 0.086 | -0.594 | -0.243 | <0.001 |
| Rodents | -0.513 | 0.062 | -0.638 | -0.388 | <0.001 |
| Large-ranged species | -0.475 | 0.056 | -0.590 | -0.361 | <0.001 |
| Small-ranged species | -0.465 | 0.076 | -0.618 | -0.311 | <0.001 |
| Endemic species | -0.526 | 0.048 | -0.626 | -0.427 | <0.001 |
| Non-endemic species | -0.355 | 0.141 | -0.646 | -0.065 | 0.010 |
